# Fasting blood glucose level and hypertension risk in aging benign prostatic hyperplasia patients

**DOI:** 10.18632/aging.102061

**Published:** 2019-07-03

**Authors:** Hao Zi, Xue-Jun Wang, Ming-Juan Zhao, Qiao Huang, Xing-Huan Wang, Xian-Tao Zeng

**Affiliations:** 1 Department of Urology, Zhongnan Hospital of Wuhan University, Wuhan, Hubei 430071, China; 2 Center for Evidence-Based and Translational Medicine, Zhongnan Hospital of Wuhan University, Wuhan, Hubei 430071, China; 3 Center for Evidence-Based Medicine, Institute of Evidence-Based Medicine and Knowledge Translation, Henan University, Kaifeng, Henan 475000, China; 4 Department of Emergency, Beijing Electric Power Hospital, Beijing 100073, China; 5 Department of Cardiology, The First Affiliated Hospital of Henan University, Kaifeng, Henan 475000, China

**Keywords:** prostatic hyperplasia, blood glucose, hypertension, diabetes mellitus, type 2

## Abstract

Evidence suggests there maybe an association among abnormal fasting blood glucose, hypertension and benign prostatic hyperplasia. In this study, we investigated whether abnormal fasting blood glucose correlates with hypertension in aging benign prostatic hyperplasia patients. Ultimately, 612 benign prostatic hyperplasia patients, including 230 hypertensive patients and 382 normotensive patients, were included. Univariate and multivariate logistic regression analyses were used to evaluate the associations. The results indicated that neither impaired fasting glucose/high risk of type 2 diabetes mellitus nor high risk of type 2 diabetes mellitus were associated with an increased risk of hypertension. When patients were stratified based on the severity of their hypertension, similar results were obtained (all *P*> 0.05). After adjusting for confounding factors, the nonsignificant tendencies for high risk of type 2 diabetes mellitus and impaired fasting glucose/high risk of type 2 diabetes mellitus to associate with hypertension persisted (all *P*> 0.05). Unlike earlier studies, the present study suggests that the level of fasting blood glucose may not be significantly related to hypertension in aging patients with benign prostatic hyperplasia.

## INTRODUCTION

Hypertension is a global problem with high morbidity. In China, the prevalence of hypertension has reached 29.6%, along with an increasing rate of obesity [1]. Metabolic syndrome is a cluster of medical conditions that includes obesity, impaired glucose metabolism, hypertriglyceridemia, low high-density lipoprotein cholesterol (HDL-C) and elevated blood pressure, which may increase the incidences of type 2 diabetes mellitus (T2DM) and cardiovascular disease [2–3]. Metabolic syndrome is known to be associated with increased risk of hypertension [4] and greater severity of benign prostatic hyperplasia (BPH) [5].

It has also been suggested that hypertension may contribute to the pathogenesis of BPH [6]. The results of a meta-analysis indicated that patients with metabolic syndrome have a significantly larger total prostate volume than those without it (+1.8 mL, 95% confidence interval [CI] 0.74–2.87; *P*<0.001) [2]. Moreover, impaired fasting glucose (IFG) may be an independent risk factor for hypertension in the Chinese [7]. Evidence indicates that the risk of hypertension is lower in females than males [8] and that lower endogenous estrogen levels in males maybe related to greater insulin resistance [9]. We therefore hypothesized that BPH may influence the relationship between IFG and hypertension. To test that idea, we investigated the association between IFG and hypertension in BPH patients. The present study was conducted and reported in accordance with the STrengthening the Reporting of OBservational studies in Epidemiology (STROBE) statement [10].

## RESULTS

### Population characteristics

Ultimately, 612 patients were included in this study. Of those, 382 were normotensive and 230 were hypertensive (155 with mild hypertension, 64 with moderate hypertension, and 11 with severe hypertension). In addition, there were 113 IFG patients and 56 at high risk of type 2 diabetes mellitus (HR-T2DM). The baseline characteristics of all participants are presented in Table 1. The mean age of all the subjects was 71.660±7.321 years. The mean ages for the normotensive and hypertensive groups were 71.196±7.292 and 72.430±7.319 years, respectively, with age being significantly lower in the normotensive group (*P*=0.043). Likewise, weight (*P*=0.002), systolic blood pressure (SBP) (*P*<0.001), diastolic blood pressure (DBP) (*P*<0.001) and hemoglobin (*P*=0.016) were all lower in the normotensive group.

**Table 1:** Baseline characteristics of the patients.

**Characteristics**	**Total**	**Normotension**	**Hypertension**	***p***
Samples	612	382	230	
Age (years)	71.660±7.321	71.196±7.292	72.430±7.319	0.043
Nationality (%)				
Han Chinese	586(95.908%)	364(95.538%)	222(96.522%)	0.552
Minority Chinese	25(4.092%)	17(4.462%)	8(3.478%)	
Marriage status (%)				
Married	3(0.491%)	2(0.525%)	1(0.435%)	1.00
Unmarried	608(99.509%)	379(99.475%)	229(99.565%)	
Height (cm)	168.032±5.727	167.930±5.721	168.206±5.747	0.581
Weight (kg)	65.553±10.906	64.437±10.114	67.388±11.895	0.002
Body mass index (kg/m^2^)	23.209±3.595	22.810±3.308	23.874±3.949	0.001
SBP (mmHg)	133.807±17.303	123.843±9.875	150.357±13.996	<0.001
DBP (mmHg)	79.946±10.771	75.372±7.705	87.543±10.858	<0.001
Resting heart rate (b/m)	76.314±9.753	75.858±9.599	77.070±9.977	0.137
t-PSA (ng/mL)	7.152±9.156	7.106±8.738	7.226±9.820	0.878
f-PSA (ng/mL)	1.358±1.442	1.290±1.220	1.469±1.747	0.187
Ratio of f-PSA/t-PSA	0.222±0.108	0.222±0.106	0.223±0.111	0.869
Prostate volume (mL)	64.644±36.170	63.805±35.833	66.062±36.769	0.459
IPSS	23.619±6.274	23.343±6.413	24.062±6.031	0.178
Comorbidities (%)				
Without	323(52.951%)	220(57.592%)	103(45.175%)	0.003
With	287(47.049%)	162(42.408%)	125(54.825%)	
Alcohol drinking status (%)				
No	284(36.835%)	180(47.120%)	104(45.217%)	0.692
Yes	94(12.192%)	55(14.398%)	39(16.957%)	
NA	393(50.973%)	147(38.482%)	87(37.826%)	
Smoking status (%)				
No	262(33.982%)	163(42.670%)	99(43.043%)	0.981
Yes	118(15.305%)	73(19.110%)	45(19.565%)	
NA	391(50.713%)	146(38.220%)	86(37.391%)	
Hemoglobin (g/L)	132.769±16.503	131.521±16.833	134.831±15.761	0.016
FBG (mmol/L)	5.395±1.486	5.413±1.656	5.364±1.153	0.667
FBG level (%)				
Normal FBG	443(72.386%)	278(72.775%)	165(71.739%)	0.853
Impaired fasting glucose	113(18.464%)	71(18.586%)	42(18.261%)	
HR-T2DM	56(9.150%)	33(8.639%)	23(10.000%)	

Data are presented as mean ± SD and percentage.

SBP, systolic blood pressure; DBP, diastolic blood pressure; FBG, fasting blood glucose; PSA, prostate-specific antigen; IPSS, international prostate symptom score; NA, not available; HR-T2DM, high risk of type 2 diabetes mellitus.

### Overall results

Table 2 summarizes the results of univariate and multivariate logistic regression analyses. In the unadjusted analysis, IFG was not associated with a greater risk of hypertension than normal fasting blood glucose (FBG) (odds ratio [OR] =0.997, 95%CI=0.650–1.528). HR-T2DM and IFG/HR-T2DM increased the risk of hypertension by 1.174 times (OR=1.174, 95%CI=0.667–2.068) and 1.053 times (OR=1.053, 95%CI=0.731–1.517), respectively, though the effect was not significant. Moreover, after adjusting for age, nationality, marriage status, body mass index (BMI), total prostate specific antigen (t-PSA), prostate volume, international prostate symptom score (IPSS), resting heart rate, hemoglobin, comorbidities, and history of smoking and alcohol drinking, none of the groups showed increased effect for FBG level on hypertension (IFG: OR=0.720, 95%CI=0.426-1.217; HR-T2DM: OR=0.804, 95%CI=0.417–1.548; IFG/HR-T2DM: OR=0.750, 95%CI=0.484–1.162).

**Table 2:** Logistic regression to explore association between levels of fasting blood glucose and degree of hypertension.

**Model**	**Comparison**	**Degree of hypertension**	**OR (95% CI)**	***P***
Unadjusted	IFG vs. Normal	Overall	0.997(0.650–1.528)	0.987
		Mild vs. Normotension	NA	0.978
		Moderate vs. Normotension	1.305(0.676–2.522)	0.428
		Severe vs. Normotension	0.962(0.590–1.568)	0.876
	HR-T2DM vs. Normal	Overall	1.174(0.667–2.068)	0.578
		Mild vs. Normotension	1.872(0.388–9.035)	0.435
		Moderate vs. Normotension	1.605(0.694–3.709)	0.269
		Severe vs. Normotension	0.961(0.488–1.892)	0.908
	IFG/ HR-T2DM vs. Normal	Overall	1.053(0.731–1.517)	0.781
		Mild vs. Normotension	0.594(0.126–2.795)	0.510
		Moderate vs. Normotension	1.400(0.797–2.458)	0.241
		Severe vs. Normotension	0.961(0.630–1.466)	0.855
Adjusted	IFG vs. Normal	Overall	0.720(0.426–1.217)	0.220
		Mild vs. Normotension	NA	0.707
		Moderate vs. Normotension	0.963(0.418–2.218)	0.930
		Severe vs. Normotension	0.702(0.386–1.274)	0.244
	HR-T2DM vs. Normal	Overall	0.804(0.417–1.548)	0.514
		Mild vs. Normotension	1.154(0.193–6.907)	0.876
		Moderate vs. Normotension	1.330(0.499–3.541)	0.568
		Severe vs. Normotension	0.600(0.271–1.328)	0.208
	IFG/ HR-T2DM vs. Normal	Overall	0.750(0.484–1.162)	0.198
		Mild vs. Normotension	0.301(0.056–1.617)	0.162
		Moderate vs. Normotension	1.092(0.551–2.165)	0.802
		Severe vs. Normotension	0.664(0.400–1.102)	0.113

OR, odds ratio; CI, confidence interval; NA, not applicable; HR-T2DM, high risk of type 2 diabetes mellitus; IFG, impaired fasting glucose.

Adjusted factors: Age, nationality, marriage status, body mass index, total prostate-specific antigen, prostate volume, international prostate symptom score, resting heart rate, hemoglobin, comorbidities, history of smoking and alcohol drinking.

### Subgroup analyses

Table 2 also shows the results of subgroup analyses, taking into consideration hypertension severity (mild, moderate, and severe groups). In the univariate analysis, IFG increased the risk of moderate hypertension by 1.305 times (95%CI=0.676–2.522). HR-T2DM increased moderate hypertension risk by 1.605 times (95%CI=0.694–3.709) and mild hypertension risk by 1.872 times of (95CI=0.388–9.035). And IFG/HR-T2DM increased moderate hypertension risk by 1.400 times (95%CI=0.797–2.458). When adjusting for confounding factors, there tended to be associations between moderate hypertension and HR-T2DM (OR=1.330, 95%CI=0.499–3.541), between moderate hypertension and IFG/HR-T2DM (OR=1.092, 95%CI=0.551–2.165), and between mild hypertension and HR-T2DM (OR=1.154, 95%CI=0.193–6.907). However, none of the results from the univariate and multivariate logistic regression analyses reached statistical difference (*P*>0.05).

## DISCUSSION

This study is based on 612 BPH patients, including 230 hypertensive and 382 normotensive patients, investigated FBG levels and hypertension risk in BPH. Our findings indicated that IFG/HR-T2DM and HR-T2DM show a tendendcy to associated with an increased risk of hypertension. After stratification based on hypertension severity, IFG, HR-T2DM and IFG/HR-T2DM all tended to associate with an increased risk of moderate hypertension, while HR-T2DM also tended to associate with an increased risk of mild hypertension. After adjusting for potential confounders, the tendency for an association between HR-T2DM and IFG/HR-T2DM and moderate hypertension and between HR-T2DM and mild hypertension persisted. However, none of these relationships were statistically significant (all *P*> 0.05).

Hypertension is an important risk factor for cardiovascular disease and is a mortality risk among the elderly [1]. In DM patients, moreover, hypertension correlates with higher risks of total mortality and cardiovascular events [11]. Previous studies have suggested that there is a link between DM and hypertension [4, 12] and that DM and hypertension are consistently predictive of the presence and severity of multiple diseases [13–14]. Three patterns of interaction (synergy, counteraction and noninterference) exist between DM and hypertension, and a possible mechanism may involve the following scenario. As FBG levels increase, hyperglycemia with insulin resistance, excessive weight and metabolic disorder may alter the renin-angiotensin in system, thereby raising blood pressure [15]. One recent study showed that, in men, high triglyceride levels, hyperglycemia, and overweight status were all associated with hypertension [16]. Similarly, higher BMIs were associated with elevations in blood glucose and mid-blood pressure [17]. We therefore adjusted for BMI, resting heart rate, hemoglobin and comorbidities in our analyses.

IFG/DM and hypertension are considered to be risk factors for BPH [5–6, 18], while elderly patients with BPH maybe more likely to also have hypertension and/or cardiovascular disease [19]. Hypertension is reportedly associated with increased expression of vascular endothelial growth factor (VEGF) within the prostatic stroma [20], and diabetic vascular damage may lead to prostatic hypoxia and the occurrence of BPH [21]. Taken together, these observations suggest the pathogenesis of BPH may involve vascular smooth muscle cell growth and remodeling as well as in prostatic smooth muscle proliferation. It is well known that IFG/DM, hypertension and BPH all share several risk factors, including age, occupation and inflammation. This is noteworthy, as the majority of symptomatic BPH patients require surgical treatment [19], which makes it important that attention is paid to the influences of hypertension and HR-T2DM/ DM on the efficacy and safety of the surgery.

Consistent with earlier findings [22–26], we observed that BPH patients with HR-T2DM or IFG/HR-T2DMhadan increased risk of hypertension. In our study, however, the associations did reach statistical significance. Three possible explanations are as follows. First, hormone changes related to BPH may affect the relationship between HR-T2DMand hypertension by affecting their progression and/or severity. In addition, the medicines used to treat BPH may also have an effect on HR-T2DM and/or hypertension. Second, BPH does not actually affect the correlation between IFG/HR-T2DM and hypertension; instead, the results presented may reflect an insufficient sample size. Because statistical power is strongly influenced by sample size, more solidly grounded case-control studies and meta-analyses are based on larger sample sizes [27]. Third, earlier studies indicate that the risk of hypertension in females is lower than in males [8], and lower endogenous estrogen levels in males maybe related to greater insulin resistance [9]. The effects in our population may thus be less pronounced because the patients were all male.

There are several limitations to our study. First, as mentioned, the sample size was insufficient to draw a strong conclusion, and there may be false positive or negative results. For example, because of the small sample size, only 11 patients with severe hypertension were recruited. We were therefore unable to fully analyze the effects of different levels of hypertension severity. Second, due to insufficient original data, we only adjusted for age, nationality, marriage status, body mass index, total prostate-specific antigen, prostate volume, international prostate symptom score, resting heart rate, hemoglobin, comorbidities, and history of smoking and alcohol drinking in this study. One or more other confounding factors may have influenced the results. For example, the absent information includes the medication history of BPH patients, which could potentially contribute to the lack of significance of the association between FBG and hypertension. In addition, we only collected data on BMI, blood pressure and FBG; metabolic syndrome status was not evaluated due to the lack of data on triglycerides and HDL-C. Third, the most recommended study design is the prospective cohort type. Our study was a retrospective analysis, which somewhat weakens our findings.

In summary, the results of the present study suggest there is a nonsignificant tendency toward a correlation between IFG/HR-T2DM and hypertension in BPH patients. Although the results were not significant, the observed tendency may help clinicians identify patients with high risk of developing hypertension when receiving the BPH treatment.

## METHODS

### Study design and patients

The study subjects were selected from the Bladder Cancer and Benign Prostatic Hyperplasia Study in Chinese Population (BPSC), which was described previously [28–30]. As of January 2018, 771 BPH patients were enrolled in the BPSC database. All eligible patients were ultimately divided into hypertensive and normotensive groups. This study was reviewed and approved by the Committee for Ethical Affairs of the Zhongnan Hospital of Wuhan University at Wuhan City, Hubei Province (Approval No. 2016028). All participants signed written informed consent forms before enrollment.

### Measurements and data

Detailed medical histories and physical examination results were obtained from all included patients. The following baseline data from blood sample examinations were recorded from the first visit of the patients: age (years), weight (kg), height (cm), marital status, nationality, DBP (mmHg), SBP (mmHg), resting heart rate (b/min), hemoglobin, FBG (ng/mL), t-PSA (ng/mL), free-PSA (ng/mL), IPSS, history of alcohol drinking and cigarette smoking, prostate ultrasonography for prostate volume (transrectal or transabdominal), and comorbidities (including high-risk hypertension, history of cerebrovascular accident, coronary heart disease, chronic bronchitis, pulmonary heart disease, severe anemia, atrial fibrillation, chronic nephritis, DM, emphysema, hepatitis B, heart failure, old pulmonary tuberculosis and gout, among others). The patients’ symptoms were assessed using IPSS, and IFG was assessed in fasting patients to determine FBG.

IFG in this study was defined as a blood glucose level ≥5.60 mmol/L (100.80 mg/dL) and <7.00 mmol/L (126 mg/dL); a FBG level >2.8mmol/L (50.40 mg/dL) and <5.60 mmol/L was defined as normal FBG (4.00-5.59 mmol/L23), and HR-T2DM was defined as a FBG level > 7.00 mmol/L (126 mg/dL) with one of the other indexes (e.g., age ≥ 40 years) from the national clinical practice guideline in China (2017 version), which was developed by the Chinese Diabetes Society [31–34]. Hypertension was diagnosed based on the SBP and DBP, or according to the patient’s medical hypertension history. Hypertension in this study was defined as an office sitting SBP no less than 140 mmHg or sitting DBP no less than 90 mmHg; hypertensive patients were then classified into mild, moderate or severe hypertension groups. The weight and height of each participant were measured, and BMI (kg/m^2^) was calculated as the weight in kilograms divided by height in meters squared. Prostate volume was calculated using the prostate ellipsoid formula [prostate volume =0.52×H (cm)×W (cm)×L (cm)] after measuring the largest anteroposterior (height, H), transverse (width, W), and cephalocaudal (length, L) prostate diameters.

### Statistical analysis

The patients were classified into two groups (0 for the normotensive group and >0 for the hypertensive group), and the statuses of the hypertensive participants were presented as four levels, from healthy to severe. FBG levels were graded as normal, IFG and HR-T2DM, and were further categorized into normal and abnormal groups. Basic characteristics were examined in the overall population and subgroups. Categorical variables were shown as counts (percentage) while continuous variables were shown as the mean ± standard deviation (SD). Comparisons of continuous variables between the normotensive and hypertensive groups were made using t-tests for continuous variables and chi-squared tests or Fisher exact test for categorical variables.

Logistic regression was implemented to explore the potential relationship between FBG level and hypertension level, and Ors with their corresponding 95% CIs were calculated. Binary logistic regression was conducted with normotensive and hypertensive groups as a dependent outcome. Ordinal logistic regression was performed for the four ordinal levels of hypertension. In these models, the effects of two types of FBG estimation was done: polytomous (normal, IFG and HR-T2DM) and binary (normal and abnormal). The confounders adjusted for in models included age, BMI, t-PSA, IPSS, resting heart rate, marital status, history of alcohol drinking and cigarette smoking, complicating disease (yes or no), and hemoglobin level. Missing values for the drinking and smoking status were coded as unknown in multiple models. Two forest plots were constructed to clearly represent the results.

Sensitivity analysis was done to determine the robustness of the results by performing nominal logistic regression. All statistical analyses were done using SPSS 19.0 software. Statistically significant tests were two-sided, and a value of P < 0.05 was considered significant.
